# Development and validation of CORe-MBC: a pragmatic measure of participatory care for adults with metastatic breast cancer

**DOI:** 10.3389/fonc.2026.1706985

**Published:** 2026-09-07

**Authors:** Carolyn Hagler, Sheetal M. Kircher, Guisselle Wismer, Wei Huang, Katherina Hauner, Stephanie Batio, Cosmina Hogea, Andrea N. Curry, Gregory Vidal, Stacy C. Bailey

**Affiliations:** 1 Feinberg School of Medicine, Northwestern University, Chicago, IL, United States; 2 Gilead Sciences, Foster, CA, United States; 3 West Cancer Center and Research Institute, Germantown, TN, United States

**Keywords:** health information technology, metastatic breast cancer, participatory care, patient engagement, patient reported outcomes measure

## Abstract

**Introduction:**

Existing research has linked increased patient participation in oncology care to greater patient satisfaction, enhanced quality of life, and improved healthcare utilization among patients with cancer. Yet, current measures of patient engagement and similar constructs do not reflect the unique experiences of patients with metastatic breast cancer; they also have limitations in terms of modality, cost, and potential for integration into clinical care. To address this, we sought to develop a new measure of participatory care in metastatic breast cancer care, referred to as the Capacity, Opportunity, and Resources in Metastatic Breast Cancer (CORe-MBC) that could be easily used in clinical care.

**Methods:**

A comprehensive pool of candidate items for CORe-MBC was created with input from MBC patients, oncologists, and experts in measure development. Items were tested among 130 English-speaking patients with MBC recruited from a large academic oncology center; participants also completed validated measures of health activation, self-efficacy, satisfaction with care, and social support (e.g., emotional, informational, and tangible). Psychometric testing included exploratory factor analyses, using oblique rotations to evaluate factor structure, and parallel analysis to test for dimensionality.

**Results:**

Patients’ ages ranged from 34 to 90 years. The majority (82%) identified as White, one-third (34%) had less than a college degree, and almost all (99%) were female. Candidate items loaded onto three factors (all factor loadings >.5), reflecting the domains of capacity, opportunity, and resources. A total of 10 items were selected for the final measure. Items demonstrated high internal consistency (α = 0.83). Domains of capacity, opportunity, and resources demonstrated moderate to high construct validity with comparable measures of patient activation (r = 0.56, p <.0001) and self-efficacy (r = 0.31–0.40, p < 0.0005), and multiple measures of satisfaction with healthcare (r = 0.42–0.55, p <.0001), and social support (r = 0.52–0.75, p <.0001), respectively.

**Conclusion:**

The CORe-MBC measure appears to be psychometrically valid and reliable. Additional studies are ongoing to further validate use of the tool among diverse populations and within a variety of oncology practices, with the goal of providing clinicians with actionable insights to tailor and improve care.

## Introduction

Over the past two decades, a growing body of research has linked increased patient participation in their oncology care to greater patient satisfaction, enhanced quality of life (QoL), and improved healthcare utilization among patients with cancer (1–3). Yet studies also show that patients with metastatic breast cancer (MBC) are less engaged in care and less likely to be to be prioritized by clinical care teams compared to patients with earlier stage breast cancer (4, 5). They also remain underrepresented in research on healthcare quality and survivorship (6, 7). As advances in oncology treatments have prolonged survival – in some cases to years or even decades – care for MBC patients has become more complex, often requiring treatment decisions that balance QoL and survival (8). As a result, improving how MBC patients and their clinical teams engage with one another to achieve high-quality, patient-centered care has become critical.

Patient reported outcomes measures (PROMs), or standardized self-assessments of how a patient is feeling and functioning, have become ubiquitous in oncology care, with results provided to clinicians to guide care (1, 2). Use of PROMs is associated with enhanced patient-provider communication, improved symptom management, and greater treatment adherence; as such, several clinical guidelines recommend their use (1, 9–11). Yet while many PROMs exist assessing physical functioning, mental health, and QoL, measures of participatory care, or the degree to which patients feel they have the capacity, opportunity, and resources needed to manage their cancer, are lacking, particularly for MBC patients who often face unique healthcare challenges and care decisions (5). Embedding a measure of participatory care into routine practice could help identify MBC patients who are struggling to engage in their cancer care, pinpoint the types of challenges encountered, and help health systems better align support and resources.

In response, our team sought to develop a new measure of participatory care in metastatic breast cancer care, referred to as the Capacity, Opportunity, and Resources in Metastatic Breast Cancer (CORe-MBC). We strived to create a measure that was brief, suitable for diverse oncology care settings, and easily integrated into clinical practice. Results were designed to provide actionable guidance to care teams that could inform targeted efforts to improve oncology care for MBC patients.

## Materials and methods

The development and evaluation of the CORe-MBC was achieved in three key phases: (a) Concept Identification, (b) Item Generation, and (c) Psychometric Testing. All study procedures for these phases were approved by the Northwestern University Institutional Review Board (IRB).

### Concept identification

Our first step was to detail the concept of participatory care for MBC and to finalize the goals of the CORe-MBC measure. Our team appreciated the existing research supporting the complementary concepts of patient engagement and patient activation, but believed currently available measures of these attributes did not necessarily reflect the distinct needs and experiences of adults living with metastatic disease and had often been underutilized or understudied in this context (12–15). Many measures of these constructs also have limitations in terms of modality, cost, and potential for integration into clinical care, a key goal of our instrument. Finally, these concepts tend to focus on individual-level skills and abilities, with less attention paid to how health system and external factors influence patient participation in care.

In consultation with a Patient Advisory Board of women with MBC who were diverse by age, race, and diagnosis, and a Scientific Advisory Board of oncologists from academic and community cancer centers across the United States, we further refined the concept of participatory care for MBC. We identified three main components we believed were critical to this concept: (a) an individual’s *capacity*, or the knowledge, skills, and motivation needed to participate in breast cancer decision-making and treatment; (b) *opportunity*, or the availability of an inclusive healthcare environment in which clinicians listen to patients and seek to optimize their MBC care; and (c) *resources*, or the external factors and support enabling patients to manage their MBC effectively, in line with their goals.

### Item generation

Candidate items for the measure were then generated through an informal literature review of published complementary measures, collaboration with the Patient Advisory Board and Scientific Advisory Board, and consultations with psychometricians and experts in measure development, who provided methodological expertise on the design and content of items. Consultations and Board meetings were iterative in nature. Multiple meetings were held via videoconferencing software over the course of 6 months, with emails used to provide study updates and hold additional discussions between meetings.

Through these processes, 70 candidate items were generated across all three domains. These items were then tested in cognitive interviews among 25 adults with MBC. Cognitive interviews were designed to explore how well items were understood and interpreted by patients with MBC and to identify those that resonated most strongly with the target population. Participants were recruited from one large academic health center using an electronic health record (EHR) query to identify potentially eligible patients (by MBC diagnosis, age 18 or older, English-speaking). Research staff contacted patients by phone, verified eligibility, engaged participants in the informed consent process, and completed one 45-minute interview with them via a secure, videoconferencing platform. During this interview, participants were asked to read and interpret potential items out loud and to offer suggestions for their improvement, if applicable. Based on participant feedback, we removed 25 items from further consideration. Remaining items were again presented to the Patient Advisory Board and Scientific Advisory Board for any comments or revisions.

### Candidate item selection and testing

After the final stage of review, 45 candidate items remained for psychometric testing; 20 of these items assessed ‘capacity,’ 16 measured ‘opportunity,’ and nine considered ‘resources’ (see **Appendix A**). Research activities for this phase were conducted from February to July 2024 and are described below.

### Study participants

For psychometric testing, patients from oncology clinics affiliated with one large academic health center in Chicago, Illinois and the surrounding suburban areas were recruited. To be eligible, patients had to: (a) be age 18 or older, (b) speak English or Spanish, (c) have a diagnosis of metastatic breast cancer in the EHR, (d) have an appointment with a oncology provider within the past 6 months, and (e) have access to a phone and the internet. Patients with any severe, uncorrectable vision, hearing, or cognitive impairment that would preclude study participation or consent were excluded from the study.

### Data collection

Participants were initially identified via an EHR query; the study team confirmed MBC diagnoses via chart review. Trained staff then contacted potentially eligible patients by phone to describe the study, determine interest, and confirm eligibility. Research staff engaged eligible and interested patients in the informed consent process and enrolled them in the study.

Participants completed one phone survey and one online survey. Surveys included measures of sociodemographic characteristics, depression (Patient-Reported Outcomes Measurement Information Systems; PROMIS) (16, 17), self-efficacy (Communication and Attitudinal Self Efficacy Scale; CASE-Cancer) (18), patient engagement (Consumer Health Activation Index; CHAI) (19), social, informational and instrumental support (PROMIS) (20), health literacy (21), and experiences with healthcare (Consumer Assessment of Healthcare Providers and Systems; CAHPS) (22). Participants were also asked to fill out an online survey that included the 45 CORe-MBC candidate items. If participants were unable to complete the online survey, it was administered verbally by study staff. Participants were compensated $40 for their time.

### Psychometric analyses

Psychometric analyses were performed to select items for the CORe-MBC measure. First, empirical reliability of all items was estimated using Cronbach’s alpha, and stability of the correlations among all candidate items was evaluated by deriving bootstrapped estimates (100 iterations) of the 95% confidence intervals around the inter-item correlations. Next, factorability was confirmed by applying Bartlett’s test of sphericity (*p* = <.001), followed by the more conservative Kaiser-Meyer-Olkin (KMO) test (overall MSA = 0.83). Exploratory factor analyses of the item variables were performed, using oblique rotations to evaluate factor structure. These analyses were conducted with items in all three domains (i.e., capacity, opportunity, and resources) separately and collectively to assess evidence for dimensionality in these item subsets and as a whole. To assess dimensionality, a scree plot and parallel analysis using Lubbe’s method compared the empirically observed eigenvalues to eigenvalues obtained from a matrix of random data.

To confirm the items selected for the final instrument models and to examine convergent validity, we used Pearson’s correlations to test agreement between the new tool’s three domains (e.g., capacity, organization, and resources) and existing measures of these and similar constructs. Four potential selections of items were considered and underwent psychometric testing (described below). Empirical reliability was again estimated using Cronbach’s alpha. All psychometric analyses were performed using R 4.2.2.

## Results

### Study sample

Overall, we attempted to contact 700 participants identified from the initial EHR query. We were unable to reach 161 patients, 297 were ineligible for the study, 108 declined to participate, and 134 consented and enrolled in the study. Of these, 132 completed the phone interview and 130 completed the online survey, which included the candidate items. The mean age of participants was 63 years. Most participants were female (>99%) and White (81%), and two-thirds (66%) had at least a college degree (see **Table 1**). Most participants had adequate health literacy, self-efficacy, and patient engagement (**Table 1**).

**Table 1:** Demographic and clinical characteristics of patient sample (*N* = 130).

Variable	*N*	%
Demographics		
Age (years), Mean (SD)	130	62.9 (12.1)
Gender		
Male	1	<1%
Female	129	>99%
Race		
Black	12	9%
White	105	81%
Asian	5	4%
Hispanic	5	4%
Education (highest level completed)		
High school or lower	12	9%
Some college or technical school	32	25%
College graduate	39	30%
Graduate school or higher	47	36%
MBC Diagnosis		
First Diagnosed		
less than 6 months	11	8%
6 to 11 months ago	13	10%
1 year to 2 years ago	14	11%
More than 2 years ago	92	71%
Early Stage		
Right away	50	39%
Progression	77	61%
MBC Type		
Don’t know	62	48%
Hormone-receptor positive AND HER2 negative	35	27%
Hormone-receptor positive AND HER2 positive	16	12%
Hormone-receptor negative AND HER2 positive	7	5%
Hormone-receptor negative AND HER2 negative	8	6%
Other	1	1%

### Item selection

Of the 45 items tested, we removed 25 from further consideration due to poor performance in the psychometric analysis. Elimination was based primarily on factor loading; however, readability, potential item overlap, clinical relevance, and prior input from patients and clinicians were all also considered by the study team. For an item to be included, a factor loading greater than or equal to 0.5 was required. With the remaining options, we assessed four models of potential item combinations. Models were devised to include three to four items per domain (e.g., capacity, opportunity, and resources), to cover the breadth of experiences with MBC described to us by our Patient and Clinician Advisory Boards, and to be brief (no more than 12 items). As all four models performed comparably, with items in the models having high factor loadings (>.5 and in most cases >.7; **Table 2**), we selected the model with the fewest items (*k* = 10; **Appendix B**) to promote ease-of-use in clinical care.

**Table 2:** Performance of CORE-MBC items (*k* = 10) in a three-factor solution.

Item	Factor number	Factor loading*
My doctor encourages me to ask questions.	1	0.8
My doctor would help me find answers to my questions.	1	0.8
My doctor spends time making sure I understand my treatment options.	1	0.8
I am confident I can stick with my treatment plans for as long as they work.	2	0.7
I am confident I can make the best health decisions for me.	2	0.7
I am confident I can find the information I need to take care of my health.	2	0.7
I have someone who will take care of me if I am not feeling well.	3	0.9
If I need help, I have someone who will help me manage my breast cancer treatment.	3	0.9
If I need help, I have someone who will help me with my daily responsibilities.	3	0.7
I have someone I can talk to about my breast cancer.	3	0.5

*All factor loadings denote positive relationships between items and underlying factors.

### CORe-MBC performance

For the selected model, the overall range of CORe-MBC scores was 28–50; the average score for items was 4.47 (*SD* = 0.50, scale 1 to 5). Reliability was high (Cronbach’s *α* ≥ 0.83). Patient age was not correlated with CORe scores (*p* = .59), nor was level of education (*p* = .29) or time since diagnosis suggesting that the model would work well across these characteristics.

The CORe capacity domain demonstrated moderate construct validity with all CASE-Cancer self-efficacy subscales, which measured patients’ ability to understand and participate in care, maintain a positive attitude, and obtain health information (*r* = 0.31–0.40 for all subscales, *p* <.0005) and health activation (*r* = 0.56, *p* <.0001; **Table 3**). The CORe opportunity domain demonstrated moderate construct validity with CAHPS satisfaction with healthcare subscales, which assess how well the cancer care team communicates with patients, patient rating of cancer care providers, and patient ratings of overall cancer care (; *r* = 0.42–0.55 for all subscales, *p* <.0001). The CORe resources domain demonstrated moderate to high construct validity with PROMIS measures of emotional, tangible, and instrumental social support (*r* = 0.52–0.75 across all three measures, *p* <.0001). Finally, the summed score of all items in the CORe-MBC was inversely associated with patient-reported depression symptoms (PROMIS Depression measure; *r* = -0.20, *p* <.03).

**Table 3:** Construct validity: psychometric properties of the CORe-MBC domains.

CORe-MBC	Comparator scale	*r* (Pearson)	*t*	*p*	*n*
Capacity^a^	CASE-Cancer Understand and participate in care^d^	0.31	3.71	.0003	130
Capacity^a^	CASE-Cancer Maintain a positive attitude^d^	0.37	4.50	.00002	130
Capacity^a^	CASE-Cancer Seek and obtain information^d^	0.40	5.01	<.0001	130
Capacity^a^	Consumer Health Activation Index^e^ (CHAI)	0.56	7.51	<.0001	128
Opportunity^b^	CAHPS How well the cancer care team^f^ communicates	0.55	7.36	<.0001	130
Opportunity^b^	CAHPS Patient rating of the cancer care team^f^	0.51	6.66	<.0001	130
Opportunity^b^	CAHPS Patient rating of overall cancer care^f^	0.42	5.25	<.0001	130
Resources^b^	PROMIS Emotional Support^g^	0.52	6.83	<.0001	130
Resources^b^	PROMIS Informational Support^g^	0.59	8.36	<.0001	130
Resources^b^	PROMIS Instrumental Support^g^	0.75	12.83	<.0001	130
Total score^c^	PROMIS Depression^h^	-0.20	-2.24	.027	128

^a^ Higher scores indicate higher confidence;.

^b^ Higher scores indicate better agreement;

^c^ Higher total scores indicate better engagement;

^d^ Communication and Attitudinal Self-Efficacy scale for cancer (CASE-Cancer). Domain scores range from 4 to 16, with higher scores indicating better confidence in communication and attitude-related self-efficacy Consumer Health Activation Index (CHAI);

^e^ Higher CHAI scores (0-100) indicate better activation (CASE-Cancer);

^f^ Consumer Assessment of Healthcare Providers and Systems. Higher domain scores indicate better assessments.

^g^ Patient-Reported Outcomes Measurement Information System (PROMIS). Higher scores indicate greater perceived support.

^h^ Patient-Reported Outcomes Measurement Information System (PROMIS). Higher scores indicate more severe depressive symptoms.

## Discussion

We developed and tested a pool of items designed to measure the degree to which MBC patients feel they have the capacity, opportunity, and resources needed to actively participate in their cancer care. From this pool, we created the CORe-MBC, a reliable, valid measure of participatory care with three key domains (e.g., capacity, opportunity, and resources). These domains demonstrated construct validity with measures of patient activation and self-efficacy, satisfaction with healthcare, and social support, respectively. A summed score of the 10 items was also significantly and inversely associated with patient-reported depressive symptoms. This is consistent with research that shows higher patient participation in care is associated with better quality of life or overall well-being; while these constructs are unique from one another, both are linked to fewer depressive symptoms (23, 24).

Importantly, this brief instrument was specifically designed to be integrated into diverse clinical practice settings, with actionable results that could inform oncology care. In particular, we theorized that the use of domains could help target intervention efforts. For example, patients who indicate challenges with ‘resources’ may be directed to a social worker for assistance while those who report difficulty related to ‘capacity’ may need additional education to understand their condition and treatment options. Conversely, a provider-facing intervention may be warranted if patients indicate challenges within the ‘opportunity’ domain; research shows providing oncologists with direct feedback on their communication skills can improve future patient counseling (25). Optimally, the measure would be deployed on a routine basis to track whether intervention efforts have made a positive impact on participatory care and whether any new challenges have arisen, with the ultimate goal of improving patient engagement in care and the care experience.

The exact ‘pathways’ for action and referral for CORe-MBC will likely need to be tailored to individual oncology practices for successful integration into clinical care (26, 27). We recently implemented this measure in multiple diverse clinical practices associated with a large academic health center in Chicago, IL and a community oncology center in Memphis, TN and found that optimal processes for delivering and responding to measure results varied. For example, while all practices in our study elected to deliver the CORe-MBC to patients electronically, some opted to have their social work team review and act upon results while others preferred to have results sent to a nurse pool for initial review. Further, the specific actions taken by these individuals in response to patients’ CORe-MBC results were tailored by site based upon available clinic and local community resources. A growing body of literature has examined how to optimally implement PROMS into clinical practices and can inform initial efforts to integrate the CORe-MBC into clinical care, although additional research specific to this measure and patient population will likely be needed (2, 27–29).

There are limitations to this research that should be noted. First, our study sample consisted of mostly white, English-speaking, higher-educated women from Chicago, Illinois. This affects the generalizability of our study results to other MBC patient populations. As mentioned above, we are now testing the instrument among patients from diverse socioeconomic backgrounds and regions of the United States to further validate the measure. We also developed a Spanish version of the CORe-MBC in tandem with the English version to promote use among linguistically diverse populations; this will need to be validated in future testing. Second, this study used a cross-sectional design. As such, we are unable to infer causal relationships and are only able to describe significant associations between CORe-MBC scores and patient outcomes like depression. Longitudinal studies are needed to better demonstrate the relationship between CORe-MBC and health behaviors and outcomes as well as to track how this construct may change over the course of patients’ MBC treatment.

The CORe-MBC joins a growing library of PROMs, which are increasingly being used in oncology settings to guide and improve clinical care (1, 2). Several systematic reviews have linked the use of PROMs to improved symptom management, health-related QoL, and overall survival in patients with cancer, including those with breast cancer (1, 30–32). Yet fewer PROMs are developed and validated for use among patients with MBC, who differ from those with early-stage breast cancer in key ways. First, patients with MBC are often focused on maintaining quality of life and delaying disease progression rather than seeking a cure; questionnaires referring to ‘future health,’ or ‘health solutions’ may not resonate with this population (33, 34). Second, greater disease complexity, higher symptom burden, and existential distress disproportionately affect MBC patients and may be inadequately captured not only in PROMs content, but also in their design and length, which can be overly burdensome (27, 34, 35). Finally, many measures of patient activation focus solely on the patient and their role in care without considering external support or resources. Recent studies have highlighted the critical importance of caregivers and support systems to engagement in care and treatment decision-making for MBC patients (36, 37).

In summary, the CORe-MBC measure appears to be a valid, reliable assessment of participatory care in adults with MBC that was designed for integration and use in oncology practices. Ongoing work by our team is examining implementation of the measure in clinical practice and further validating the tool in socioeconomically diverse populations. Results should provide further information on the generalizability of the tool and its feasibility of use in clinical practice.

## Data Availability

The raw data supporting the conclusions of this article will be made available by the authors, without undue reservation.

## Appendix

**Appendix A:** Items included in psychometric testing.

Capacity
1. I am confident I can access my health records online.
2. I am confident I can ask my doctor questions about my breast cancer.
3. I am confident I can ask my doctor questions about my treatment options.
4. I am confident I can be honest with my doctor about what I want.
5. I am confident I can change my treatment plan if I need to.
6. I am confident I can decide what treatment options are best for me.
7. I am confident I can decide when to seek a second opinion.
8. I am confident I can decide when to seek care if something seems wrong.
9. I am confident I can find the information I need to take care of my health.
10. I am confident I can follow through with any treatment plans I make with my doctor.
11. I am confident I can involve family members in healthcare discussions as much or as little as I would like.
12. I am confident I can make and keep healthcare appointments.
13. I am confident I can make the best health decisions for me.
14. I am confident I can manage my emotions well enough to take care of my breast cancer.
15. I am confident I can participate in discussions about my care, even when I get bad news.
16. I am confident I can reach out to my doctor if I am having a problem with my treatment.
17. I am confident I can reflect on what is important to me at this time in my life.
18. I am confident I can stick with my treatment plans for as long as they work.
19. I am confident I can take an active role in discussions with my doctor.
20. I am confident I can take an active role in managing my health

Opportunity
1. My doctor always listens to what I say.
2. My doctor and I have talked about my preferences for treatment.
3. My doctor asks me if I have any treatment concerns.
4. My doctor cares about my wellbeing.
5. My doctor encourages me to ask questions.
6. My doctor explains the treatment options for my breast cancer in a way I understand.
7. My doctor explains things in a way that I understand.
8. My doctor gives me clear instructions for my treatment.
9. My doctor is knowledgeable about my breast cancer.
10. My doctor is knowledgeable about my treatment options.
11. My doctor spends time making sure I understand my treatment options.
12. My doctor supports me making my own healthcare decisions
13. My doctor takes time to ask me about my treatment preferences.
14. My doctor wants to know me as a person.
15. My doctor would help me find answers to my questions.
16. My doctor would support me if I wanted to get a second opinion.

Resources
1. I have someone I can talk to about my breast cancer.
2. I have someone who will take care of me if I am not feeling well.
3. I have what I need to access my health records online.
4. If I need help, I have someone who will help me manage my breast cancer treatment.
5. If I need help, I have someone who will help me with my daily responsibilities.
6. If I need help, I have someone who will take me to health care appointments.
7. It is hard for me to pay for my breast cancer treatment.
8. My life keeps me from going to my healthcare appointments.
9. I can always get to my healthcare appointments.

**Appendix B:** Final core MBC tool.

The purpose of this questionnaire is to understand how confident and supported you feel discussing and making decisions about your breast cancer. This questionnaire has 3 sections and should take about 5 minutes to complete.
The first set of questions are about how confident you feel managing your breast cancer. Please rate how confident or not confident you feel with each of the following statements.
Capacity
1. I am confident I can stick with my treatment plans for as long as they work.
o Extremely confident o Very confident o Somewhat confident o Not very confident o Not confident at all
2. I am confident I can make the best health decisions for me.
o Extremely confident o Very confident o Somewhat confident o Not very confident o Not confident at all
3. I am confident I can find the information I need to take care of my health.
o Extremely confident o Very confident o Somewhat confident o Not very confident o Not confident at all
Opportunity
The next set of questions are about how well your doctor at [Insert Clinic Name] involves you in discussions about your breast cancer. Please rate how strongly you agree or disagree with each of the following statements.
4. My doctor encourages me to ask questions.
o Strongly agree o Agree o Neither agree nor disagree o Disagree o Strongly disagree
5. My doctor would help me find answers to my questions.
o Strongly agree o Agree o Neither agree nor disagree o Disagree o Strongly disagree
6. My doctor spends time making sure I understand my treatment options.
o Strongly agree o Agree o Neither agree nor disagree o Disagree o Strongly disagree
Resources
The last set of questions are about support available to you at home from friends, family members, or other caregivers. Please rate how strongly you agree or disagree with each of the following statements.
7. I have someone who will take care of me if I am not feeling well.
o Strongly agree o Agree o Neither agree nor disagree o Disagree o Strongly disagree
8. If I need help, I have someone who will help me manage my breast cancer treatment.
o Strongly agree o Agree o Neither agree nor disagree o Disagree o Strongly disagree
9. If I need help, I have someone who will help me with my daily responsibilities.
o Strongly agree o Agree o Neither agree nor disagree o Disagree o Strongly disagree
10. I have someone I can talk to about my breast cancer.
o Strongly agree o Agree o Neither agree nor disagree o Disagree o Strongly disagree
